# Oral Contraceptive Use and Breast Cancer Risk for BRCA1 and BRCA2 Mutation Carriers: Systematic Review and Meta-Analysis of Case–Control Studies

**DOI:** 10.3390/cancers14194774

**Published:** 2022-09-29

**Authors:** Agnieszka Barańska, Wiesław Kanadys

**Affiliations:** 1 Department of Medical Informatics and Statistics with E-Learning Lab, Medical University of Lublin, 20-954 Lublin, Poland; 2 Medical University of Lublin, 20-090 Lublin, Poland

**Keywords:** BRCA1, BRCA2, breast cancer, oral contraceptives, case–control study

## Abstract

**Simple Summary:**

The aim of the present work was to systematically review and meta-analysis the available evidence regarding the effects of oral contraceptives using on breast cancer risk in BRCA germline mutations. The included studies were published between 2002 and 2021. Data were pooled from nine case–control studies, comprising a total of 33,162 woman. The association between oral contraceptive use and risk of breast cancer may differ in breast cancer defined by BRCA mutation status. This meta-analysis showed a diverse effect of oral contraceptive use against breast cancer in BRCA carrier mutations. However, futher case control studies are necessary to examine breast cancer risk.

**Abstract:**

Oral contraceptive use is one of the major modifiable risk factors for breast cancer. To investigate the effect of oral contraceptive taking on breast cancer risk by BRCA 1 and BRCA 2 mutation status, we conducted a systematic review and meta-analysis of case-controlled studies. Therefore, English language articles were retrieved by searching MEDLINE (PubMed), EMBASE and the Cochrane Library up to August 2021. Data were pooled from none case–control studies, comprising a total of 33,162 subjects, including 23,453 who had never used oral contraceptives. Overall meta-analysis indicated a statistically insignificant risk reduction: OR = 0.86, 95% CI: 0.70 to 1.06, *p* = 0.1594. However, increased breast cancer risk was associated with age at first use of OCs ≥20 years: OR = 1.21, 95% CI:1.07 to 1.36, *p* = 0.002. Multivariable meta-regression with covariates of age of first OC use (β = 0.21, 95% CI: −0.25 to 0.67, *p* = 0.3767), duration of OC use (β = −0.08, 95% CI; −0.51 to 0.34, *p* = 0.7093), and time since last OC use (β = 0.32, 95% CI: −0.22 to 0.85, *p* = 0.2461) did not have a significant effect on the breast cancer risk. This meta-analysis suggests a diverse effect of oral contraceptive use against breast cancer in BRCA carrier mutation. The association between OC use and breast and ovarian cancers needs more investigation.

## 1. Introduction

Breast cancer genetic, heterogeneous diseases are characterized by high variability by ethnicity and race in terms of incidence, clinical features, and prognosis. In the general population, breast cancer is the leading cancer type in females, comprising 11,7% of all new cancer cases and causing 6.9% of all cancer-related deaths worldwide in 2020 [1,2]. The majority of breast cancer cases are sporadic; however, it is estimated that approximately 5% to 10% of these are linked to genetic disorders [3]. Most frequently, a strong genetic predisposition to breast cancers is related to mutations in high penetrance tumor suppressor genes: BRCA1 (BReast-CAncer 1) and BRCA2 [3,4]. This predisposition is also detected in a series of genes coding for proteins that interact with BRCA1 or BRCA2 or act in the same DNA repair pathway. Inherited mutations in these genes predispose to breast cancer with varying penetration estimates. 

The genes with high penetrance include the following genes: TP53, CDH1, PTEN, and STK11; the rare genes with medium penetrance are CHEK2, ATM PALB2, and BRIP1 [5]. PALB2 is characterized as an important predisposing to breast cancer gene after BRCA1 and BRCA2, despite the fact that it is classified as a gene with moderate penetrance [6].

Genes of BRCA1 and BRCA2 are localized on chromosomes 17q21 and 13q12, respectively [7,8]. Both BRCA 1 and BRCA2 genes protect the genome from damage, at least in part by repairing DNA and regulating transcription in response to DNA damage or by maintaining the stability of chromosomes, regulating key mitotic steps or cell division, and regulating other important cellular processes [9,10].

The complete loss of function of both proteins leads to an increase in genomic instability. Germline mutations in these genes increase the risk of many malignancies over the course of life, especially breast cancer. The risk of developing breast cancer in women with BRCA mutations ranges from 45% to 75%. Among BRCA1 mutation carriers, the estimated lifetime risk of developing breast cancer ranges from 40% to 85%. In the case of the BRCA2 mutation, carriers have more or less the same risk of developing breast cancer [4,11,12,13]. 

The frequency of germline BRCA1/2 gene mutation carriers and the ratio of germline BRCA1 to BRCA2 mutations in BRCA-related breast cancer patients vary depending on the population [14]. BRCA pathogenic mutations occur at earlier ages, the mean age being 43 years at diagnosis [15]. Breast cancer risk clearly increases with an increased number of first- and second-degree relatives diagnosed as having breast cancer for both BRCA1 and BRCA2 carriers [4].

Among BRCA carriers, data are limited on the influence of modifying factors on breast cancer risk. In both BRCA mutations, early onset menarche increases breast cancer risk; first birth after age 30 y reduces breast cancer risk in BRCA1 but generates increased risk in BRCA2, while breastfeeding decreases breast cancer risk in both BRCA1 and BRCA2 mutation carriers [3,15,16,17]

In addition to the genetic risk factors, as well as the mentioned risk modifying factors, endocrine interventions should be mentioned. Among these, one of the most common is oral contraception (OC) [18]. In the general population, oral contraceptives use leads to a significantly increased risk for breast cancer [19,20]. However, whether exogenous estrogens, such as oral contraceptives, modify the cancer risk in BRCA1/2 mutation carriers is a controversial topic. Data and evidence relating to this dependency are still contradictory; some studies suggest that oral contraceptives may increase the risk of cancer, while others show no effect [21,22,23]. 

The aim of the present work was to systematically review and meta-analysis the available evidence regarding the effects of oral contraceptives using on breast cancer risk in BRCA germline mutations.

## 2. Materials and Methods

This systematic review with meta-analysis was designed according to PRISMA (The Preferred Reporting Items for Systematic Reviews and Meta-analysis) guidelines to identify case–control studies examining the effect of oral contraceptive use on breast cancer risk in mutation carriers in BRCA1 and BRCA2 [24].

### 2.1. Search Strategy and Selection Criteria

We reviewed the contents of the electronic bibliographic databases: PubMed (MEDLINE), EMBASE, and the Cochrane Library for articles compatible with the assumptions of our work. The search was limited to papers published in English and was conducted up to August 2021. In the computer search, we used the following keywords in various combinations: (‘breast cancer’) AND (‘BRCA1′ OR ‘BRCA2′) AND (‘oral contraceptives’ OR ‘birth control pill’). To provide a complete overview of the available relevant publications, for this purpose, we additionally scrutinized references to previously published review articles, meta-analyses, and other works not retrieved in our electronic search.

Studies were considered eligible for inclusion if they met the following criteria: the study describes outcomes among BRCA1/2 mutation carriers; of case–control design; it provides data on the quantitative association between OC use and risk of breast cancer; healthy mutation carriers were the control group; data included in the articles were sufficient to calculate the odds ratio (OR) and 95% confidence interval (CI); and the articles were written in English. The exclusion criteria were as follows: no control group; the control group was not mutation carriers; lack of sufficient information; results were reported as graphics or percent changes; duplicated reports; and articles were published in languages other than English. Inclusion/exclusion decisions were made after independent and duplicate examination of the full manuscripts.

### 2.2. Data Extraction and Quality Assessment

Data were extracted from all eligible studies by the lead author and subsequently reviewed by the co-author for accuracy. The information consisted of the first author’s name, year of publication, region of the study, study design, number of cancer cases, number of controls, and characteristics of the studied populations.

We assessed and scored the methodological quality of included studies based on three aspects according to the Newcastle–Ottawa Scale (NOS), that is, study design (including the selection of study population), data comparability, and outcome assessment [25]. On a scale from zero to nine, studies scored five or greater were considered to be of high quality, while those scored below five were classified as low quality.

### 2.3. Statistical Analysis

The meta-analysis of summary statistics from individual studies was carried out using the STATISTICA 13.3 program (StatSoft Polska, Kraków, Poland) by way of employing the Medical Package program. Based on the data obtained from each study, we calculated the odds ratio (OR) and the 95% confidence interval, a cross-classification of OC users and breast cancer type. A meta-analysis was carried out combining OR from various studies using the DerSimonian–Laird random effects model [26]. In addition, a pre-planned subgroup analysis was performed by the age of first contraceptive use (<20 years vs. ≥20 years), duration of contraception (≥5 years compared to <5 years), and years from the last use of the contraceptive prior to diagnosis (<10 years in compared to ≥10 years). We used I^2^ and Cochrane Q to evaluate heterogeneity, by the following criteria: high heterogeneity, I^2^ >75%; moderate heterogeneity, I^2^ = 50% to 75%; and low heterogeneity, I^2^ < 50% [27]. 

The risk of publication bias was assessed by applying Egger’s linear regression test [28] and Begg’s rank correlation test [29]. We also determined the possibility of publication bias by visually checking the asymmetry of the funnel plot. In the absence of bias, the plots resemble a symmetrical funnel, as the results of minor studies scatter on the left side of the plot, and the spread narrows among the major studies on the right side of the plot [30]. 

## 3. Results

**Figure 1:**
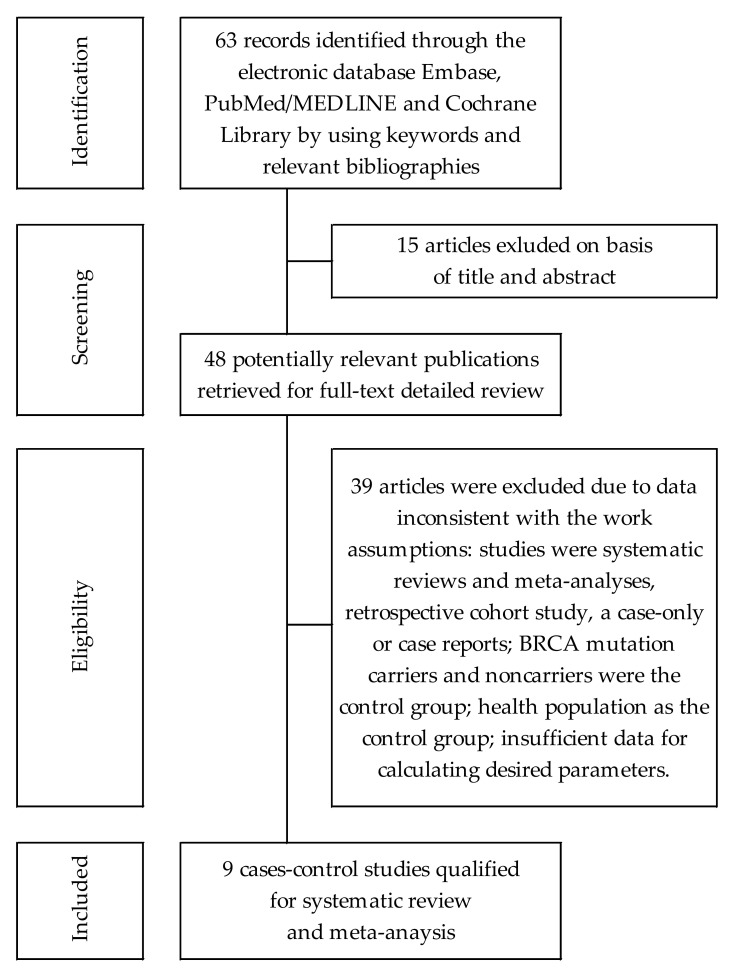
Flowchart of the selection procedure for studies included in the current review and meta-analysis.

A detailed review of selection procedures is shown in Figure 1. Overall, 63 articles were found after a multi-database search. After reviewing their titles and abstracts, 48 full-text articles containing potentially useful data were included for further evaluation. Of these, nine articles were finally qualified for meta-analysis [31,32,33,34,35,36,37,38,39]. 

The included studies were published between 2002 and 2021. Data were pooled from nine case–control studies, comprising a total of 33,162 subjects, including 23,453 (70.7%) who had never used oral contraceptives (OCs). Characteristics of the included clinical trials are shown in Table 1. The group of cases (breast cancer) included 14,342 women, of which 9748 (68.0%) women were using OCs. For comparison, in the control group (unaffected BRCA mutation carriers), there were 18,820 women, including 13,705 (72.8%) women who used OCs. The studies provided data for BRCA1/2 mutation carriers (eight trials), BRCA1 mutation carriers (six trials), and BRCA2 mutation carriers (four trials).

The quality of the analyzed studies as assessed on the basis of the Newcastle–Ottawa Scale ranged between 5 and 8, and the average score was 6.22 for included studies.

### 3.1. Oral Contraceptives and Breast Cancer in BRCA1/2 Mutation Carriers

**Figure 2:**
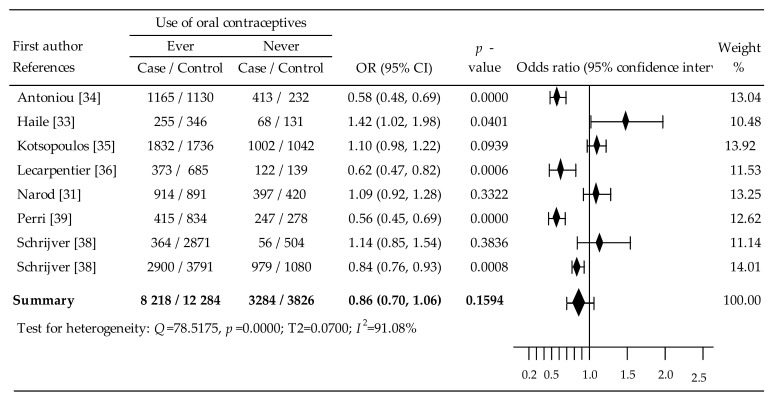
F Forest plots for association between oral contraceptives and breast cancer in BRCA1/2 mutation carriers [31,33,34,35,36,38,39]. Note: data are expressed as mean differences with 95% confidence intervals (CIs), using random effects models; effect is represented by the block diamond; the horizontal lines denote the 95% CIs, some of which extended beyond the limits of the scale.

Eight comparisons of data [31,33,34,35,36,38,39] contributed to meta-analysis on the effects OC on breast cancer risk in BRCA1/2 mutation carriers (Figure 2). Compared to control groups, a statistically significant reduction in breast cancer risk was notated in four studies [34,36,38,39]; in four studies, an increase in risk was observed, including where in one study, change was statistically significant [33]. Overall meta-analysis indicated an insignificant reduction: OR = 0.86, 95% CI: 0.70 to 1.06, *p* = 0.1594. The major problem of this analysis was the high heterogeneity rate (I^2^ = 91.08%). The Egger’s test (b_0_ = −1.2052, 95% CI: −1.20, 95% CI: −8.9554 to 6.5449, *p* = 0.7167), however, indicated no evidence of publication bias, while Begg’s test (Tau-b = 1.0000, z = 2.0381, *p* = 0.0415) suggested possible publication bias. In the subgroup analysis, only age at first use of OCs ≥20 years revealed a significant increase in breast cancer risk (OR = 1.21, 95% CI:1.07 to 1.36, *p* = 0.002). 

Multivariable meta-regression with covariates of age of first OC use (β = 0.21, 95% CI: −0.25 to 0.67, *p* = 0.3767), duration of OC use (β = −0.08, 95% CI: −0.51 to 0.34, *p* = 0.7093), and time since last OC use (β = 0.32, 95% CI: −0.22 to 0.85, *p* = 0.2461) did not have a significant effect on the breast cancer risk.

### 3.2. Oral Contraceptives and Breast Cancer in BRCA1 Mutation Carriers

The influence of OCs on the risk of breast cancer in BRCA1 mutation carriers was analyzed on the basis of six trials [32,33,34,37,38]. In three of them, a risk reduction was observed [32,34,38], including a statistically significant decrease in two trials [34,38]. A non-significant increase in the risk of breast cancer was observed in three trials [33,37,38].

**Figure 3:**
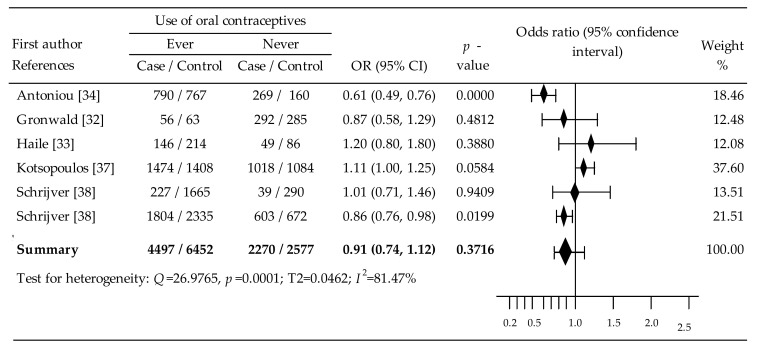
Forest plots for association between oral contraceptives and breast cancer in BRCA1 mutation carriers [32,33,34,37,38]. Note: data are expressed as mean differences with 95% CIs, using random effects models; effect is represented by the block diamond; the horizontal lines denote the 95% CIs, some of which extended beyond the limits of the scale.

The meta-analysis of all the included studies noted insignificant reduction in breast cancer risk: OR = 0.91, 95% CI: 0.74 to 1.12, *p* = 0.3716 (Figure 3) and relatively moderate heterogeneity was shown (I^2^ = 79.36%). Publication bias was not evident according to the Begg’s test (Tau-b = 0.6667, Z = 1.3587, *p* = 0.1742) and Egger’s test (b_0_ = −0.7563, 95% CI: −6.5074 to 4.9948; *p* = 0.7335). 

A subgroup analysis of the relationship between the risk of breast cancer in BRCA1 mutation carriers and the age of the first OCs application was performed on the basis of data from four citations [33,37,38]. Starting OCs intake <20 years was associated with a slight increase in the risk of breast cancer, OR = 1.02, *p* = 0.88, while intake of OCs ≥ 20 years led to a statistically significant increase in the risk of breast cancer: OR = 1.28, *p* = 0.02. There was also a statistically significant reduction in the risk of breast cancer in BRCA1 mutation carriers based on the variable comparing of starting intake of OC at the age below vs. above 20 years; OR = 0.78, *p* = 0.0002 (Table 2).

We also carried out an analysis of dependencies between breast cancer risk in BRCA1 mutation carriers and years since the last use of OCs prior to diagnosis, based on four citations [33,37,38]. The last OCs use age in a period of less than 10 y before diagnosis was associated with a statistically significant reduction in breast cancer risk: OR = 0.84, *p* = 0.009. In turn, the last use of OCs ≥ 10 y was associated with a marginal, insignificant increase in risk: OR = 1.08, *p* = 0.39. The relationship between the risk of breast cancer in BRCA 1 mutation carriers and the covariate <10 years vs. ≥10 years was a statistically significant reduction: OR = 0.83, *p* = 0.002 (Table 2). Other results in subgroup analyses based on the mentioned above pre-specified factors did not reveal a significant effect on breast cancer risk (Table 2).

Multivariable meta-regression with covariates of age first use of OCs (β = 0.22, 95% CI: −0.14 to 0.57, *p* = 0.2227), duration use of OCs (β = 0.06, 95% CI: −0.34 to 0.22, *p* = 0.6960), time since last use (β = 0.21, 95% CI: −0.03 to 0.45, *p* = 0.0815) showed these covariates had non-significant impact on breast cancer risk.

### 3.3. Oral Contraceptives and Breast Cancer in BRCA2 Mutation Carriers

**Figure 4:**
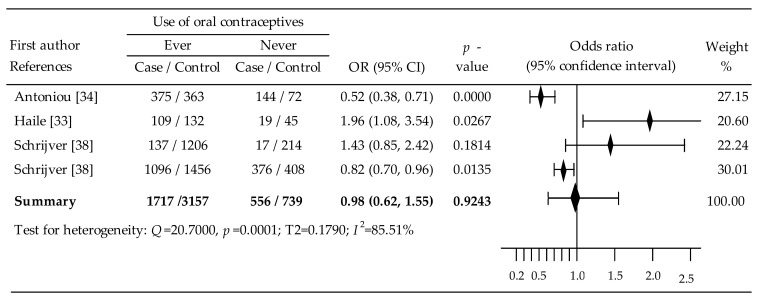
Forest plots for association between oral contraceptives and breast cancer in BRCA2 mutation carriers [33,34,38]. Note: data are expressed as mean differences with 95% CIs, using random effects models; effect is represented by the block diamond; the horizontal lines denote the 95% CIs.

The relationship between OC administration and the risk of breast cancer in BRCA2 mutation carriers was assessed in four trials [33,34,38]. Two studies demonstrated a statistically significant reduction in the risk of breast cancer [34,38]. One study found a statistically significant increase in the risk of breast cancer [33], and one study found the increase in risk not significant [38]. The random-effects meta-analysis showed a slight, non-significant reduction in the risk of breast cancer: OR = 0.98, 95% CI: 0.62 to1.55, *p* = 0,9243; with relatively high heterogeneity: I^2^ = 85.51% (Figure 4). The result of Begg’s test was inaccessible, while Egger’s test (b_0_ = –0.7335, 95% CI: –10.1208 to 14.6620; *p* = 0.5131) did not show evidence of publication bias (Table 2).

Based on data from three citations [33,38], an analysis of the influence of age of first OCs use on the risk of breast cancer in BRCA1 mutation carriers was performed. Accordingly, starting their use ≥20 years resulted in a statistically significant increased risk of OC, OR = 1.42, *p* = 0.03. In turn, in the analysis of covariate <10 years vs. ≥10 years for years since last use of OCs prior to diagnosis, a statistically significant reduced risk: OR = 0.65, *p* = 0.000, I^2^ = 0.00% (Table 2). We found that multivariate meta-regression with covariates of age of first OC use (β = 0.26, 95% CI: –0.56 to 1.08, *p* = 0.5396), duration of OC use (β = –0.01, 95% CI: –0.66 to 0.63, *p* = 0.9693), and time since last OC use (β = 0.40, 95% CI: –0.48 to 1.27, *p* = 0.3736) did not have a significant effect on the risk of breast cancer.

## 4. Discussion

Overall meta-analysis for BRCA1/2 indicated an in-significant risk reduction: OR = 0.86, 95% CI: 0.70 to 1.06, *p* = 0.1594. In the subgroup analysis, only age at first use of OCs ≥20 years revealed a significant increase in breast cancer risk (OR = 1.21, 95% CI:1.07 to 1.36, *p* = 0.002). The meta-analysis in BRCA1 mutation carriers noted insignificant reduction in breast cancer risk: OR = 0.91, 95% CI: 0.74 to 1.12, *p* = 0.3716. Intake of OCs ≥20 years led to a statistically significant increase in the risk: OR = 1.28, *p* = 0.02. The relationship between the risk of breast cancer in BRCA mutation carriers and the covariate <10 years vs. ≥10 years was a statistically significant reduction: OR = 0.83, *p* = 0.002. The random-effects meta-analysis in BRCA2 mutation carriers showed a slight, non-significant reduction in the risk of breast cancer: OR = 0.98, 95% CI: 0.62 to1.55, *p* = 0,9243. Starting OC use ≥20 years resulted in a statistically significant increased risk: OR = 1.42, *p* = 0.03. In turn, in the analysis of covariate <10 years vs. ≥10 years, a statistically significant reduced risk of breast cancer was noted: OR = 0.65, *p* = 0.000.

In this systematic review and meta-analysis, we incorporated evidence gathered in recent studies that taking oral contraceptives may influence the risk of breast cancer in BRCA mutation carriers. Although the results show no statistical significance, our meta-analysis suggested the need for prospective, controlled studies on extensive material of BRCA mutation carriers regarding the use of modern oral contraceptives. Whether exogenous estrogens, such as oral contraceptives, modify the breast cancer risk in BRCA1 and BRCA2 mutation carriers is actually a controversial topic. Some studies suggest that oral contraceptives may increase the breast cancer risk among BRCA mutation carriers [34,36,38,39]; others reported only a little or no influence of oral contraceptives on breast cancer risk [31,32]. A large study conducted by Brohet et al. [40] found that the use of oral contraceptives, as well as the longer duration of oral contraceptive use, were not only associated with an increased breast cancer risk but also with an earlier onset. Moorman et al. [41], based on five studies published from 2000 to 2012, showed a non-statistically significant increased risk among OC users (BRCA1: OR, 1.19; BRCA2: OR 1.36; BRCA1/2: OR = 1.21). In a systematic review regarding the relationship between OC use and breast cancer risk, Huber et al. [42] took into account four meta-analyses, one review, one case–control study, two case-only studies, one prospective, and one retrospective cohort study. Herein, some studies reported a risk elevation, while others did not find an association between OC use and breast cancer in BRCA mutation carriers. In other studies, the association was limited to early onset breast cancer and/or associated with young age at the first start of OC. 

There are several limitations that should be considered when interpreting the data. The results of the meta-analysis are based on a relatively limited number of available studies, as well as on different numbers of participants and variable observation time in individual samples, which may result in insufficient statistical power and limit the drawing of final conclusions. Moreover, the different periods of research carried out are associated with the use of different doses of contraceptive preparations and different ingredients. Furthermore, the characteristics of the women participating in the studies may also have an impact with regard to various comorbidities, as well as inter-individual differences in the metabolism and bioavailability of OC. Overall, data on the risk of OC use in BRCA mutation carriers are limited. Almost all of the available studies are retrospective, and especially for BRCA2 mutation carriers, study populations were often small. We are aware that one of the limitations of our meta-analysis is the use of repeated data, to a varying degree, from different research periods provided by the co-authors of these studies in individual multicenter studies.

Women at moderate risk of breast cancer have several options to reduce their risk, including lifestyle options, i.e., physical activity, BMI control, and no alcohol consumption [43]. High BMI in postmenopausal years is associated with a significant increase in breast cancer risk [44,45]. A study showed that regular alcohol consumption is the leading modifiable cause of breast cancer burden for premenopausal women, explaining 12.6% of breast cancer [46]. Furthermore, physical activity is associated with about a 20% reduced risk of breast cancer when compared the most with the least physically active women [47]. 

However, for women who have an increased risk of developing breast cancer, there are additional factors that can change and reduce the risk of breast cancer, including surgery and medication [43,48]. Tamoxifen and raloxifene block the effects of estrogen in the breast tissue, and aromatase inhibitors treatments that aim to lower the estrogen levels can also be a solution for postmenopausal women [48,49,50]. Aromatase inhibitors reduce recurrence rates by about 30%, and aromatase inhibitor reduces 10-year breast cancer mortality rates by about 15% compared with 5 years of tamoxifen, hence by about 40% compared with no endocrine treatment [51].

## 5. Conclusions

The association between oral contraceptive use and risk of breast cancer may differ in breast cancer defined by BRCA mutation status. This meta-analysis showed a diverse effect of oral contraceptive use against breast cancer in BRCA carrier mutations. However, this association needs more investigation. 

## Figures and Tables

**Table 1:** Characteristics of case–control studies on the association between breast cancer risk and oral contraception use among BRCA mutation carriers.

Autor Pub Year [Ref.]	Study Name Setting	Study Year	Study Populations of Mutation Carriers Cancer N (n, %) Unaffected N (n, %)	NOS Scale
Narod et al. 2002 [31]	International study 11 countries ^a^	1977–2001	Cases: 1311 *BRCA1/2* (69.7) Controls: 1311 *BRCA1/2* (68.0)	5
Gronwald et al. 2006 [32]	Poland	1988–2005	Cases: 348 BRCA1 (16.1) Controls: 348 BRCA1 (18.1)	8
Haile et al. 2006 [33]	Australia, Canada, USA and Utah		Cases: 195 *BRCA1* (74.9); 128 *BRCA2* (85.2) Controls: 302 *BRCA1* (70.9);179 *BRCA2* (73.4)	6
Anatoniou et al. 2009 [34]	IBCCS 15 countries ^b^	1997–2005	Cases: 1100 *BRCA1* (71.2); 531 *BRCA2* (70.6) Controls: 970 *BRCA1* (79.1); 455 *BRCA2* (79.8)	6
Kotsopoulos et al. 2012 [35]	International 5 countries ^c^		Cases: 2584 BRCA1/2 (64.6) Controls: 2584 BRCA1/2 (63.3)	7
Lecarpentier et al. 2012 [36]	GEBESPO France	2000–2010	Cases: 499 BRCA1/2 (74.7) Controls: 838 BRCA1/2 (81.7)	8
Kotsopoulos et al. 2014 [37]	HBCCSG 13 countries ^d^		Cases: 2,492 *BRCA1* (59.2) Controls: 2,492 *BRCA1* (56.5)	
Schrijver 2018 [38]	EMBRACE, BCFR, IBCCS, kConFab, Othere 14 countries ^e^		a. Prospective cohort: Cases: 2,544 *BRCA1* (71.5); 1,560 *BRCA2* (70.9) Controls: 3,163 *BRCA1* (77.4); 1,965 *BRCA2* (73.9) b. Retrospective full cohort: Cases: 269 *BRCA1* (84.0); 157 *BRCA2* (86.6) Controls: 2,007 *BRCA1* (82.7); 1.453 *BRCA2* (82.7)	8
Perri et al. 2021 [39]	Israel	1995–2019	Case: 687 *BRCA1/2* (64.0) Controls: 1,137 *BRCA1/2* (75.5)	8

Note: ^a^ United States, Canada, Israel, Poland, Netherlands, Norway, Italy, U.K., Austria, Sweden, France; ^b^ Austria, Belgium, Germany, Netherlands, Hungary, Poland, Denmark, Sweden, France, Italy, Canada (Quebec), Spain, United Kingdom and Eire; ^c^ United States, Canada, Israel, Poland, Austria; ^d^ USA, Canada, Poland, Israel, Netherlands, Norway, Italy, France, Austria, Sweden, United Kingdom, China, Bahamas; ^e^ USA, Germany, U.K., Netherlands, France, Canada, Australia, Spain, Austria, Czech Republic, Hungary, Denmark, Sweden, Poland. Abbreviations: BCFR, Study, and the Breast Cancer Family Registry; EMBRACE, Epidemiological Study of Familial Breast Cancer; GENEPSO, National BRCA1, and BRCA2 mutations carrier cohort; HOCCSG, Hereditary Ovarian Cancer Clinical Study Group; IBCCS, International BRCA1/2 Carrier Cohort Study; kConFab, Kathleen Cuningham Foundation Consortium for Research Into Familial Breast Cancer Follow-Up Study; NOS, Newcastle–Ottawa Scale, OC, oral contraceptive; N, number of participants; n, percentage of ever OC use.

**Table 2:** Pooled estimates of effect of taking oral contraceptives on breast cancer risk.

Subgroup	n	OR	95% CI	*p*	I*^2^* (%)	Begg’s Test			Egger’s Test			
Outcoms						Tau-b	Z	*p*	b0	95% CI	T	*p*
***BRCA1/2* carriers mutations**												
Oral contraceptives (OCs) use [31,33,34,35,36,38,39]												
Ever	8	0.86	0.70 to 1.06	0.159	91.08	1.000	2.038	0.041	−1.205	−8.955 to 6.545	−0.380	0.717
Never	8	Referent										
Age at first use the OCs [33,38]												
<20 years	3	1.06	0.70 to 1.60	0.798	87.90	Inaccessible			4.492	−6.114 to 15.099	5.382	0.117
≥20 years	3	1.21	1.07 to 1.36	0.002	0.00	1.000	1.567	0.117	1.206	−1.0270 to 3.438	6.862	0.092
<20 years/≥20 years	3	0.81	0.60 to 1.08	0.154	77.92	Inaccessible			3.777	−6.130 to 13.684	4.844	0.130
Duration of OCs use [33,34,38]												
≥5 years	4	0.84	0.67 to 1.06	0.149	71.55	Inaccessible			2.244	−8.367 to 12.855	0.910	0.459
<5 years	4	0.94	0.67 to 1.33	0.723	90.23	Inaccessible			3.487	−12.020 to 18.994	0.967	0.435
≥5 years/<5 years	4	1.05	0.86 to 1.27	0.655	73.91	Inaccessible			3.348	−7.767 to 14.464	1.296	0.324
Years since last use of OCs prior to diagnosis [33,38]												
<10 years	3	0.92	0.65 to 1.29	0.623	80.01	Inaccessible			3.579	−0.597 to 12.755	4.956	0.127
≥10 years	3	1.27	0.84 to 1.29	0.249	85.60	Inaccessible			4.3770	−7.437 to 16.19	4.708	0.133
<10 years/≥10 years	3	0.75	0.68 to 0.83	0.000	0.00	−1.000	−1.567	0.117	−1.050	−7.761 to 5.660	−1.989	0.297
***BRCA1* carriers mutations**												
Oral contraceptives (OCs) use [32,33,34,37,38]												
Ever	6	0.90	0.75 to 1.10	0.359	79.36	0.667	1.359	0.174	−0.756	−6.507 to 4.995	−0.365	0.733
Never	6	Referent										
Age at first use the OCs [33,37,38]												
<20 years	4	1.02	0.77 to 1.35	0.880	84.45	Inaccessible			1.284	−13.214 to 15.782	0.381	0.740
≥20 years	4	1.28	1.04 to 1.57	0.019	62.00	Inaccessible			−1.420	−9.347 to 6.506	−0.771	0.521
<20 years/≥20 years	4	0.78	0.69 to 0.89	0.000	27.10	0.667	1.359	0.174	1.967	−2.548 to 6.482	1.874	0.202
Duration of OCs use [33,34,37,38]												
<5 years	5	0.85	0.70 to 1.04	0.115	67.86	0.333	0.522	0.601	−1.140	−7.653 to 5.373	−0.557	0.616
≥5 years	5	0.90	0.74 to 1.10	0.298	75.60	1.000	1.567	0.117	0.499	−7.157 to 8.156	0.208	0.849
≥5 years/<5 years	5	1.03	0.91 to 1.16	0.653	36.70	0.600	1.470	0.142	1.927	−2.732 to 6.587	1.316	0.280
Years since last use of OCs prior to diagnosis [33,37,38]												
<10 years	4	0.84	0.74 to 0.96	0.009	11.61	Inaccessible			1.740	−2.004 to 5.484	1.999	0.184
≥10 years	4	1.08	0.90 to 1.31	0.394	63.25	Inaccessible			1.326	−6.600 to 9.252	0.720	0.546
<10 years/≥10 years	4	0.83	0.73 to 0.93	0.002	11.19	−1.000	−2.038	0.041	−1.711	−6.826 to 3.403	−1.440	0.287
***BRCA2* carriers mutation**												
Oral contraceptives (OCs) use [33,34,38]												
Ever	4	0.98	0.62 to 1.55	0.924	85.51	Inaccessible			0.733	−10.121 to 14.662	0.788	0.513
Never	4	Referent										
Age at first use the OCs [33,38]												
<20 years	3	1.23	0.61 to 2.50	0.563	87.41	Inaccessible			4.192	−7.113 to 15.498	4.712	0.133
≥20 years	3	1.42	1.04 to 1.93	0.027	30.59	Inaccessible			1.892	−4.307 to 8.092	3.878	0.161
<20 years/>−20 years	3	0.76	0.50 to 1.14	0.187	73.79	Inaccessible			3.109	−20.053 to 26.272	1.706	0.338
Duration of OCs use [33,34,38]												
<5 years	4	0.93	0.63 to 1.36	0.709	71.21	Inaccessible			2.115	−7.863 to 12.094	0.912	0.458
≥5 years	4	0.98	0.59 to 1.63	0.936	86.33	Inaccessible			3.106	8.484 to 14.696	1.153	0.368
≥5 years/<5 years	4	0.94	0.74 to 1.20	0.635	56.69	1.000	1.567	0.117	3.034	−4.326 to 10.394	1.774	0.218
Years since last use of OCs prior to diagnosis [33,38]												
<10 years	3	1,00	0.51 to 1.96	0.995	83.93	Inaccessible			3.663	−14.208 to 21.534	2.604	0.233
≥10 years	3	1.46	0.83 to 2.57	0.187	77.67	Inaccessible			3.393	0.990 to 5.796	17.941	0.035
<10 years/≥10 years	3	0.65	0.55 to 0.76	0.000	0.00	0.333	0.522	0.601	0.534	−16.921 to 17.990	0.389	0.764

Abbreviations: CI, confidence interval; I^2^, coefficient of inconsistency; n, number of studies; OR, odds ratio; *p*, probability value.
