# Expression of Concern: Barańska, A.; Kanadys, W. Oral Contraceptive Use and Breast Cancer Risk for BRCA1 and BRCA2 Mutation Carriers: Systematic Review and Meta-Analysis of Case–Control Studies. *Cancers* 2022, *14*, 4774

**DOI:** 10.3390/cancers15143668

**Published:** 2023-07-19

**Authors:** Cancers Editorial Office

**Affiliations:** MDPI, St. Alban-Anlage 66, 4052 Basel, Switzerland; cancers@mdpi.com

The Cancers Editorial Office is issuing this expression of concern to alert readers to concerns regarding the findings of the published manuscript [1]. As the publication could influence patient treatment, the Cancers Editorial Office has decided to issue an expression of concern for the duration of the investigation.

The Expression of Concern will remain until a resolution has been reached.

We will provide an update following the conclusion of the investigation. The author has been notified about this Expression of Concern. In order to maintain the highest scientific standards, an in-depth investigation has been initiated by the responsible editors, together with the journal’s editorial office, in collaboration with the editorial board, and in accordance with the Committee on Publication Ethics (COPE) guidance.
